# Use of SOPARC to assess physical activity in parks: do race/ethnicity, contextual conditions, and settings of the target area, affect reliability?

**DOI:** 10.1186/s12889-019-8107-0

**Published:** 2019-12-23

**Authors:** Oriol Marquet, J. Aaron Hipp, Claudia Alberico, Jing-Huei Huang, Dustin Fry, Elizabeth Mazak, Gina S. Lovasi, Myron F. Floyd

**Affiliations:** 1 ISGlobal Barcelona Institute for Global Health, Barcelona, Spain; 2 Department of Parks, Recreation, and Tourism Management, NC State University, Raleigh, NC USA; 3 Center for Geospatial Analytics, NC State University, Raleigh, NC USA; 4 Dornsife School of Public Health, Drexel University, Philadelphia, PA USA

**Keywords:** SOPARC, Reliability, Observational, Park use, Park physical activity, Race/ethnicity

## Abstract

**Background:**

Since its introduction in 2006, SOPARC (Systematic Observation of Play and Recreation in Communities) has become a fundamental tool to quantify park visitor behaviors and characteristics. We tested SOPARC reliability when assessing race/ethnicity, physical activity, contextual conditions at the time of observation, and settings of target areas to understand its utility when trying to account for individual characteristics of users.

**Methods:**

We used 4725 SOPARC observations completed simultaneously by two independent observers to evaluate intraclass correlation and agreement rate between the two observers when trying to assess sex, age group, race/ethnicity, and level of physical activity of urban park users in different park settings. Observations were in 20 New York City parks during Spring and Summer 2017 within the PARC^3^ project.

**Results:**

Observers counted 25,765 park users with high interobserver reliability (ICC = .94; %Agreement.75). Reliability scores were negatively affected by the population being observed, the intensity of physical activity, and the contextual conditions and settings of the target area at the time of observation. Specific challenges emerged when assessing the combination of physical activity and race/ethnicity.

**Conclusions:**

SOPARC training should aim to improve reliability when assessing concurrent measures such as physical activity, race/ethnicity, age, and sex. Similarly, observing crowded park areas with many active users areas may require more observation practice hours.

## Background

Research has long demonstrated how physical activity and recreation in public open spaces are positively associated with health outcomes [1, 2]. Systematic observation studies in spaces such as parks and playgrounds, however, can be challenging because of the lack of objective measures to quantify visitor characteristics and behavior. Accurate assessment of large numbers of physically active park users poses additional challenges due to the constant dynamic movement of park users.

Since its introduction in 2006, Systematic Observation of Play and Recreation in Communities (SOPARC) [3] has provided an answer to those challenges. SOPARC is based on momentary sampling through periodic and systematic scans of delimited target areas. It provides a consistent method to count large groups of people while they are taking part in highly-dynamic activities without placing a burden on participants [4]. The development of SOPARC and its rapid adoption by researchers allowed a better understanding of the context in which physical activity occurs while being able to measure the behavior of large groups of people. Its development has allowed evaluation to move beyond individual measurements and self-reported physical activity.

Previous research shows that the interrater reliability of SOPARC observers is improved by a period of training and practice [5]. Reliability as measured by intraclass correlation when counting park users using SOPARC, for instance, has been previously reported as ranging between 0.94 and 1.0 by Chow et al. [6], as consistently greater than 0.8 by Cohen et al. [7] and by over 0.9 by Banda et al. [8]. Reliability values have been slightly lower when observers have tried to assess a combination of age and sex; age and physical activity; or age and ethnicity. Floyd et al. [9] reported reliability as low as 0.78 when counting sedentary boys, and Chung-Do et al., reported 0.44 Kappa coefficients when trying to assess boys engaging in vigorous activities. Santos et al. [10] encountered more difficulties when counting male and female teenagers than when counting male and female children. Other contextual conditions such as time of the day, day of the week, or first versus last rounds of SOPARC observation on a given day might determine observer fatigue, potentially resulting in variability in attentiveness and lower interobserver reliability. The presence of organized activities –both formal and informal- typically entail not only more people in the target area but can also involve distractions including spectators, officials, and additional equipment making the task more difficult for the observer. Finally, the particular settings of the target area being observed might also have implications for observers. Observing physical activity in a basketball court, for example, might prove harder than observing users in a swing set; the intricate design of some playgrounds can generate blind spots for observers; and assessing race/ethnicities of park users in large areas such as baseball fields can prove difficult due to distance, masks or other related paraphernalia. Even after subdividing target areas, as recommended by the SOPARC protocol, some of these problems may persist.

To date, no study has analyzed the SOPARC reliability when assessing race/ethnicity and physical activity of users of recreational spaces. And although most studies using SOPARC report some measure of general reliability, no effort has been made to understand how contextual variables at the time of the observation may affect reliability scores.

In order for SOPARC to be a useful tool, it is not possible simply to sidestep these issues. The SOPARC protocol is one of the most frequently used methods to assess park-use and understand physical activity and the demographic composition of park visitors [5]. For example, given the known racial disparities in physical activity [11, 12] access to park settings [13], and outdoor opportunities [14] in the US, being able to use the SOPARC protocol not only to assess the use of space in parks and playgrounds [15] but also to gather individual user characteristics [16] is a key goal of both leisure and public health studies. For these reasons, for the PARC^3^ project it was of prime importance to attempt to measure race/ethnicity, age, sex and physical activity at the same time, despite assuming that interrater reliability was likely to be negatively affected by the addition of more stratifying variables. Because of that, after introducing minor modifications to the original form we aimed at evaluating SOPARC reliability and its capacity to accurately assess physical activity levels among different racial/ethnic population groups.

Specifically, this study aimed to describe how reliability in observations of park users may be affected by (1) assessing race/ethnicity in combination with physical activity, (2) contextual conditions at the time of the observation, and (3) setting of the target area. Additionally, we examined how each of these factors in combination might affect interobserver reliability when using SOPARC.

## Methods

### Data collection

Data for this analysis were obtained by 4725 paired SOPARC observations in 20 New York City parks conducted during the spring and summer of 2017 within the PARC^3^ project (Physical Activity and Recreation of Children in Communities of Color [15–19]). Parks were located in low-income census blocks with high prevalence of Latino or Asian populations (for more information on park selection please see: 15). To be able to report PA at the individual level and describe individual park user characteristics, modifications were made to the original SOPARC form, which proposes sex to be an anchor variable for each scan, and age group, physical activity, and race/ethnicity to be observed separately. The adapted forms (see Additional file 1) required each scan to use both sex and age group as anchors, thereby generating 10 scans per target area per round. At each scan, race/ethnicity and physical activity were observed for the sex and age group anchored. For example, females 0–4 years old were observed first. At the time of the scan, all females apparently 0–4 years old were assessed for race/ethnicity and for physical activity level.

Seven observers were trained in 2 days for a total of 9 hours including lecture and practical field training. The observers were college-educated adults living in New York City. Given the fact that diversity of observers have shown to improve reliability scores [20], our set of observers included two men and four women from diverse backgrounds including race and ethnicity. Following conventional SOPARC protocol, targets were scanned from left to right for each sex connected to an age group. Observed variables included sex (male; female), age groups (children 0–4 years old; children 5–10; teens 11–19; adults 20–64; older adults 65+), race/ethnicity (White; Black; Asian; Latino; Other/unsure), and physical activity levels (sedentary; moderate/walking; vigorous). Because the overall study was interested in identifying use of parks by minority communities, specific emphasis was given to distinguishing between African-Americans, Asian, and Latino users. However, because of the inherent complexities of trying to identify race/ethnicity from external phenotype characteristics alone, we used the “Other” category to account for all users that could not be classified clearly within African-American, Asian, Latino or White categories. All parks were visited and SOPARC administered on two weekdays and two weekend days in both the spring and the summer, for one-hour periods (3 pm, 4:30 pm, and 6 pm during spring; 10 am and 6 pm during summer). Each observation consisted of four rounds within an hour -one every 15 min- and each round included a total of 10 scans for each target area.

These target areas (*n* = 167) that had been previously identified and geolocated by research team members included playground sets, swing sets, water features, basketball courts, baseball/handball fields and other areas [16]. Each target area was observed in pairs during a one-hour period, each round at 15 min (e.g., 3:00, 3:15, 3:30, 3:45) between April and June and July–August 2017. Study procedures were approved by the North Carolina State University Institutional Review Board (IRB#9376).

### Reliability measures

A wide range of statistical scores and metrics are available to measure reliability when observing park use. Most previous SOPARC studies have used either one or a combination of intraclass correlation and interobserver agreement [5]. The intraclass correlation coefficient (ICC) is the most widely used metric to measure observer agreement [5, 21]. ICC is an estimate of the relative magnitude of variation for the relationship between multiple assessments of the same observation as compared with variation across observations [22, 23]. Scores < 0.4 are usually interpreted as a poor agreement between the observers, with 0.4–0.75 signifying fair to good agreement, and values > 0.75 meaning excellent agreement beyond chance [24, 25]. Interobserver agreement, which evaluates the degree of concordance between assessments of two or more observers, measures the proportion of occasions that individuals gave the same score [23]. Interobserver agreement values over 70% are typically considered high [26]. Agreement values however are subject to any shared systematic biases the observers might have which might result in poor agreement even in cases with high intraclass correlation.

Because interobserver agreement is increasingly hard to achieve in crowded park areas, we followed McKenzie et al. [3] and used a variant overall proportion of agreement indicator by which in areas where at least one observer counted 11 or more people, a 10% discrepancy between observers was allowed and still counted as an agreement. We used both ICC and interobserver agreement to analyze SOPARC reliability when assessing the number of park users stratified by race/ethnicity and physical activity in specific park and contextual conditions.

Reliability scores do not make sense when areas are empty, as they will falsely represent high agreement scores. Therefore, areas with zero park users were excluded from the final database for the reliability analysis (*n* = 1335 observations excluded) following Mckenzie et al. [3] method. However, additional methodological challenges appear when trying to assess the reliability of counts aiming at specific groups, such as the number of children of a specific age range or a single race/ethnicity.

As an example, within our dataset when all available paired observations (*n* = 4725) are considered, the two observers agree on the number of Asian park users in the target area 79% of the time (Table 1). When applying the McKenzie et al. [3] correction and using only those pairs of observation where at least one person of any race was counted by either of the observers (*n* = 3390), we find the observers agree only 71% of the time. At this point, however, there might still be areas with at least one person but no Asians, and thus agreement might still be slightly inflated due to the presence of zeros. One could select only those pairs of observations where at least one Asian person was counted by either of the two observers (*n* = 1837) in which case we find that the pair of observers agreed 47% of the time on the exact number of Asians present in the target area. This restrictive method, however, is highly limiting in terms of sample size, especially when trying to compare reliability scores in terms of race/ethnicity or with physical activity. Because most SOPARC analysis either have low sample sizes [4, 27] or have only a subsample of observations actually collected in pairs [4, 6, 28], this most restrictive method seems to challenge future replicability. Because of this we chose to use all observations made in nonempty areas (*n* = 3390) as our base sample.

**Table 1:** Descriptive statistics and univariate reliability items

	Park-users	Observations	Users per observation^b^	ICC	95CI L	95CI H	% Agreement^c^
All observations							
Total	25,765	4725	5.453	0.941	0.938	0.945	75.2
Race/Ethnicity							
Asian American	9488	4725	2.008	0.929	0.925	0.933	79.4
Latino	9143	4725	1.935	0.853	0.845	0.861	62.4
African American	4701	4725	0.995	0.770	0.758	0.781	69.8
White-Caucasian	1695	4725	0.359	0.880	0.873	0.886	0.91
Other	1025	4725	0.217	0.936	0.932	0.940	0.96
Physical activity							
Sedentary	10,211	4725	2.161	0.895	0.889	0.900	63.3
Moderate	11,449	4527	2.529	0.782	0.771	0.793	54.6
Vigorous	3459	4527	0.764	0.564	0.544	0.583	67.1
Nonempty areas only^a^							
Total	25,767	3390	7.601	0.915	0.909	0.92	65.5
Race/Ethnicity							
Asian	9488	3390	2.799	0.922	0.917	0.927	71.3
Latino	9143	3390	2.697	0.828	0.817	0.838	47.6
African American	4701	3390	1.387	0.748	0.733	0.763	58.0
White-Caucasian	1695	3390	0.500	0.877	0.869	0.884	39.5
Other	1025	3390	0.302	0.934	0.930	0.939	24.8
Physical activity							
Sedentary	10,211	3390	3.012	0.878	0.87	0.886	48.9
Moderate	11,950	3390	3.525	0.712	0.695	0.728	36.7
Vigorous	3607	3390	1.064	0.517	0.492	0.541	54.2

^a^Observations in areas where at least one of the two observers counted one or more people

^b^Average of the counts reported by the two observers

^c^Percent of times where both observers counted the exact same number of people in the target area. In areas with 11 or more people, a < 10% discrepancy is still considered as agreement

### Statistical analysis

We first analyzed reliability scores of pairs of observers assessing race/ethnicity and physical activity independently (Table 1). We used paired sample t-tests to identify significant differences when trying to assess each race/ethnicity category and each physical activity intensity. We also used paired t-tests to measure how reliability scores changed when trying to identify combined measures such as the number of people of a single race/ethnicity engaging in a particular physical activity intensity (Table 2).

**Table 2:** Agreement rate differences between two observers assessing race/ethnicity and physical activity

	Obs.	Difference btw. Means^a^	t	Std. Dev.	95% CI	*p*-value
Race/ethnicity						
Asian American vs. Latino	3390	0.24	23.198	0.60	0.22; 0.26	< 0.001
Asian American vs. African American (AA)	3390	0.13	11.408	0.68	0.11; 0.16	< 0.001
Latino vs. AA	3390	−0.10	−11.362	0.53	−0.12; − 0.09	< 0.001
Physical activity						
Sedentary vs. Moderate	3390	0.12	13.322	0.53	0.1; 0.14	< 0.001
Sedentary vs. Vigorous	3390	−0.05	−4.850	0.63	−0.07; − 0.03	< 0.001
Moderate vs. Vigorous	3390	− 0.17	− 18.832	0.54	− 0.19; − 0.16	< 0.001
Race/ethnicity x physical activity						
Asian Sedentary vs. Latino Sedentary	3390	0.11	10.647	0.62	0.09; 0.13	< 0.001
Asian Moderate vs. Latino Moderate	3390	0.16	13.823	0.66	0.14; 0.18	< 0.001
Asian Vigorous vs. Latino Vigorous	3390	0.52	5.538	0.50	0.5; 0.54	< 0.001
Asian Sedentary vs. AA Sedentary	3390	0.00	0.217	0.63	−0.02; 0.02	0.828
Asian Moderate vs. AA Moderate	3390	0.02	1.918	0.69	0; 0.05	0.055
Asian Vigorous vs. AA Vigorous	3390	−0.04	−3.903	0.56	−0.06; − 0.02	< 0.001
Latino Sedentary vs. AA Sedentary	3390	− 0.11	−11.801	0.55	− 0.13; − 0.09	< 0.001
Latino Moderate vs. AA Moderate	3390	− 0.13	−13.216	0.59	− 0.15; − 0.11	< 0.001
Latino Vigorous vs. AA Vigorous	3390	− 0.09	−10.471	0.52	− 0.11; − 0.08	< 0.001

^a^Paired sample t-test comparing the rate of agreement between observers when assessing the first variable vs the second one

In addition, Chi-square tests were used to examine associations between target area settings and contextual conditions and interobserver agreement variance (Table 3). Contextual conditions included the day of the week (weekends or weekdays), time of day when the observation was taken (10-11 am; 3-4 pm; 4.30–5:30 pm; 6-7 pm), and round number (1–4). Variables concerning the settings of the target area included the amount of people present in the target area (average between the assessment of the two observers) type of activity setting (playground, basketball, baseball or handball court, swing set, water feature, or other), presence of organized activities (formal or informal), and the presence or absence of shade. We used binary logistic regression to calculate the odds of achieving agreement in the different target area characteristics.

**Table 3:** Intraclass correlation and % observer agreement by area and context characteristics

		ICC			Agreement		Adjusted agreement^b^		
	N	ICC	95CI		%	*P*^a^	*B*	*P*	95CI
Average people counted						< 0.001			
< = 5.00	1445	0.636	0.604; 0.666		81.2		Ref	–	–
5.01–15.00	1645	0.638	0.609; 0.666		53.8		0.230	< 0.001	0.10; 0.28
15.01+	300	0.706	0.644; 0.758		53.7		0.243	< 0.001	0.18; 0.32
Day of week						0.426			
Weekday	2528	0.895	0.887; 0.902		65.3		Ref	–	–
Weekend	862	0.948	0.94; 0.954		65.8		1.101	0.297	0.92;1.32
Time of day						< 0.001			
10-11 am	240	0.521	0.423; 0.608		46.0		Ref	–	–
3 pm–4 pm	1011	0.953	0.947; 0.958		71.4		3.976	< 0.001	2.89; 5.47
4.30–5.30 pm	668	0.901	0.885; 0.914		65.6		3.093	< 0.001	2.22; 4.30
6-7 pm	1471	0.948	0.942; 0.953		65.4		3.452	< 0.001	2.53; 4.71
Round						0.452			
1	1011	0.927	0.918; 0.935		63.7		Ref	–	–
2	995	0.913	0.902; 0.923		67.1		1.122	0.050	1; 1.49
3	875	0.924	0.914; 0.933		65.6		1.137	0.219	0.93; 1.39
4	509	0.878	0.857; 0.897		65.4		1.205	0.139	0.94; 1.54
Formally organized						0.149			
N	3338	0.919	0.914; 0.924		65.6		Ref	–	–
Y	52	0.644	0.454; 0.779		57.7		1.663	0.093	0.92; 3.01
Informally organized						0.228			
N	2689	0.917	0.911; 0.923		65.8		Ref	–	–
Y	701	0.899	0.884; 0.912		64.2		0.173	0.224	0.90; 1.52
Shade cover						0.032			
None	999	0.932	0.924; 0.94		64.0		Ref	–	–
Some	1285	0.914	0.905; 0.923		68.3		1.149	0.143	0.95; 1.39
Complete	1014	0.904	0.892; 0.915		63.8		1.174	0.146	0.95; 1.46
Target area						< 0.001			
Basketball	809	0.810	0.785; 0.832		61.6		Ref	–	–
Baseball/Handball	379	0.904	0.884; 0.921		69.4		0.918	0.560	0.69; 1.23
Playground	1120	0.938	0.93; 0.945		61.6		0.916	0.512	0.70; 1.19
Swings	508	0.929	0.916; 0.94		77.8		2.043	< 0.001	1.49;2.80
Water	237	0.945	0.929; 0.957		67.5		0.912	0.626	0.63;1.32
Other	337	0.951	0.94; 0.96		63.2		0.924	0.629	0.67;1.28
Total	3390	0.915	0.909; 0.92		65.5				

^a^Pearson chi-square test of association between % agreement and area characteristic

^b^Logistic regression coefficients representing odds of achieving agreement per type of observation

Finally, in Table 4 we report results on the chances of the two observers being in agreement (+ − 10%) when counting people from each race/ethnicity and physical activity categories while accounting for all contextual variables. Because the PARC^3^ study was set in parks located in census blocks with high presence of Asian, Latino and African American populations, we decided to keep Table 4 focused only on these same race-ethnicities. To do so we implemented a binary logistic regression in which we regressed the odds of agreement using the number of people in the target area, type of day, time of day, round number, presence of organized activities, presence of shade and the type of activity setting as predictors. To assist with interpretation, we present the estimated marginal effects calculated at the sample means, representing the chance of the two observers being in agreement on the exact number of people (+ − 10%) of a specific race/ethnicity present in the target area. All analysis were performed using SPSS (v25 IBM SPSS) and STATA (v15 StataCorp LLC, College Station, TX, USA) with 0.05 level of significance.

**Table 4:** Reliability metrics when assessing race/ethnicity and physical activity in distinct observation conditions

	Sedentary						Moderate						Vigorous					
	Asian *N* = 4101 43.6%		Latino *N* = 3500 40.2%		African American *N* = 1568 32.9%		Asian *N* = 4133 43.9%		Latino *N* = 3966 45.5%		African American *N* = 2372 49.8%		Asian *N* = 1171 12.5%		Latino *N* = 1247 14.3%		African American *N* = 816 17.2%	
	ICC^a^	Agr.^b^	ICC^a^	Agr.^b^	ICC^a^	Agr.^b^	ICC^a^	Agr.^b^	ICC^a^	Agr.^b^	ICC^a^	Agr.^b^	ICC^a^	Agr.^b^	ICC^a^	Agr.^b^	ICC^a^	Agr.^b^
Average people counted																		
< = 5.00	0.73	0.88	0.67	0.80	0.60	0.85	0.66	0.81	0.58	0.69	0.54	0.76	0.50	0.90	0.47	0.86	0.46	0.90
5.01–15.00	0.82	0.67*	0.72	0.54*	0.61	0.68*	0.73	0.62*	0.57	0.41*	0.64	0.60*	0.50	0.75*	0.44	0.67*	0.60	0.79*
15.01+	0.90	0.46*	0.79	0.28*	0.23	0.54*	0.76	0.41*	0.58	0.32*	0.43	0.45*	0.42	0.59*	0.41	0.54*	0.45	0.79*
Day of week																		
Weekday	0.91	0.77	0.76	0.62	0.52	0.71	0.79	0.71	0.63	0.52	0.62	0.63	0.47	0.81	0.47	0.73	0.51	0.81
Weekend	0.88	0.68*	0.81	0.66*	0.26	0.81*	0.73	0.59*	0.62	0.52	0.53	0.72*	0.53	0.76*	0.44	0.76	0.76	0.89*
Time of day																		
10-11 am	0.93	0.62	0.45	0.62	0.34	0.8	0.49	0.51	0.38	0.51	0.22	0.68	0.42	0.72	0.07	0.73	0.06	0.86
3 pm–4 pm	0.93	0.77*	0.84	0.65	0.60	0.73*	0.75	0.73*	0.62	0.56	0.66	0.64	0.66	0.83*	0.52	0.77	0.57	0.84
4.30–5.30 pm	0.91	0.75*	0.77	0.58	0.37	0.68*	0.85	0.71*	0.62	0.49	0.62	0.62	0.54	0.82*	0.42	0.73	0.42	0.81
6-7 pm	0.88	0.74*	0.76	0.64	0.55	0.76	0.8	0.65*	0.66	0.51	0.61	0.67	0.42	0.78	0.46	0.73	0.66	0.83
Round																		
1	0.89	0.74	0.80	0.63	0.46	0.74	0.75	0.67	0.62	0.53	0.61	0.65	0.60	0.80	0.55	0.74	0.51	0.83
2	0.91	0.76	0.81	0.62	0.59	0.73	0.79	0.68	0.59	0.51	0.64	0.66	0.36	0.79	0.4	0.73	0.65	0.84
3	0.92	0.73	0.71	0.64	0.62	0.76	0.79	0.67	0.68	0.53	0.57	0.65	0.57	0.79	0.45	0.74	0.53	0.83
4	0.91	0.75	0.76	0.65	0.37	0.73	0.8	0.69	0.63	0.51	0.65	0.63	0.46	0.82	0.43	0.75	0.62	0.83
Formally organized																		
N	0.90	0.74	0.79	0.63	0.50	0.74	0.78	0.68	0.63	0.52	0.61	0.65	0.50	0.80	0.46	0.74	0.58	0.83
Y	–	–	–	–	–	–	–	–	0.64	0.51	–	–	–	–	–	–	–	–
Informally organized																		
N	0.91	0.73	0.79	0.64	0.51	0.76	0.73	0.66	0.61	0.54	0.52	0.67	0.41	0.78	0.45	0.75	0.45	0.85
Y	0.76	0.79*	0.69	0.61	0.47	0.68*	0.83	0.73*	0.63	0.47*	0.67	0.59*	0.49	0.83*	0.49	0.69*	0.64	0.78*
Shade cover																		
None	0.92	0.72	0.69	0.61	0.45	0.72	0.78	0.66	0.57	0.52	0.64	0.64	0.53	0.80	0.46	0.77	0.55	0.83
Some	0.86	0.71	0.76	0.65	0.54	0.76*	0.75	0.67	0.66	0.55	0.63	0.66	0.39	0.79	0.49	0.75	0.65	0.86
Complete	0.89	0.81*	0.82	0.62	0.52	0.73	0.77	0.71*	0.62	0.49	0.58	0.66	0.46	0.8	0.45	0.69*	0.45	0.80*
Target area																		
Basketball	0.71	0.75	0.51	0.74	0.46	0.76	0.79	0.65	0.51	0.55	0.66	0.61	0.46	0.75	0.59	0.80	0.63	0.77
Baseball/Handball	0.76	0.67*	0.77	0.62*	0.44	0.91*	0.63	0.54*	0.70	0.49*	0.61	0.87*	0.19	0.71	0.29	0.69*	–	–
Playground	0.90	0.73	0.81	0.58*	0.52	0.65*	0.76	0.68	0.62	0.49	0.28	0.58	0.56	0.78	0.42	0.69*	0.35	0.80
Swings	0.84	0.82*	0.77	0.64*	0.72	0.76	0.86	0.73*	0.61	0.47*	0.52	0.57	0.60	0.87*	0.63	0.71*	0.52	0.84*
Water	0.91	0.67*	0.69	0.64*	0.30	0.71	0.74	0.71	0.62	0.63*	0.42	0.72*	–	–	0.46	0.84	–	–
Other	0.96	0.75	0.78	0.48*	0.36	0.78	0.50	0.78*	0.68	0.59	0.64	0.84*	0.66	0.91*	0.42	0.8	–	–
Total	0.90		0.78		0.49		0.78		0.63		0.61		0.5		0.46		0.58	

^a^Bivariate Intraclass Correlation Coefficient (ICC)

^b^Marginal effects of binary logistic regression calculated at the sample means of all covariates. Interobserver agreement when assessing the number of park users per each race/ethnicity and physical activity as the dependent variable

- Insufficient sample size

* α < 0.05

## Results

A total of 25,765 park users observed during the PARC3 study formed the basis for this analysis. Adequate reliability was recorded when observing race/ethnicity and physical activity separately, both when using all observations (*n* = 4725; ICC = 0.941) and only observations made in nonempty areas (*n* = 3390; ICC = 0.915) (Table 1). In nonempty areas, the agreement was high for estimating the number of Asian park users (ICC = 0.922; Agreement = 71.3%), and low for estimating the number of people engaging in vigorous physical activity (ICC = 0.517; Agreement = 54.2%).

Paired-samples t-tests were used to evaluate if there were significant differences in the rate of agreement between observers when assessing race/ethnicity and physical activity (Table 2). Observers agreed more often when counting Asian Americans than Latinos (t = 23.2 *p* < 0.001), or African Americans (t = 11.41 *p* < 0.001), and they agreed less often when counting African Americans than Latinos (t = − 11.36 *p* < 0.001). In terms of physical activity, agreement was higher when assessing sedentary activity than moderate activity (t = 13.32 *p* < 0.001), but less than assessing vigorous activity (t = − 4.85 *p* < 0.001). When trying to assess both race/ethnicity and physical activity jointly, the higher discrepancies were found when comparing reliability achieved when counting Asian Americans vigorous activity to the reliability at counting Latinos’ vigorous activity (t = 5.538 *p* < 0.001).

When contextual conditions and settings of the target area were taken into account (Table 3) we found a decrease in the rate of agreement when more than five people were observed in the target area. There were however no major differences in interobserver reliability when observing areas with 5–15 people, as compared with more than 15 people. While the day of week or round of SOPARC did not appear to affect the rate of interobserver agreement, observing in the morning seemed to be less reliable than observing at any other time of the day. In terms of settings of the target area, having organized activities did not compromise the reliability of the observation. Interobserver agreement however was slightly lower when observing completely shaded or completely sunny areas, and also when observing basketball courts and playgrounds. The adjusted agreement rates, that account for the characteristics of each target area, confirm that agreement is harder to achieve in areas with more than 5 people, and easier to achieve in swing areas, in comparison with basketball courts.

Finally, Table 4 shows results of combining the reliability variance due to characteristics of the observed population and the variance due to contextual conditions and the settings of the target area. When counting sedentary Asian Americans, the lowest ICC was found when observing basketball courts (0.71). In the case of African American sedentary counts, lowest reliability was found when observing water features (0.3) or other types of areas (0.36). Bivariate coefficients however do not adjust by other covariates that might be affecting the observation reliability. To provide a more accurate assessment of reliability, the estimated marginal means of regressing the overall interobserver agreement for each race/ethnicity and physical activity are presented also in Table 4. The marginal means show that reliability at assessing all ethnicity and physical activity groups is always higher when observing areas with fewer people (< 5) than target areas with greater number of park users. Reaching agreement on the number of Asian American park users was significantly more difficult on weekends (sedentary: 68%; moderate: 59%; vigorous: 76%) than weekdays (sed: 77%; mod: 71%; vig: 81%). Conversely, when observing African Americans, better agreement was reached on weekends (sed: 81%; mod: 72% vig: 89%) than on weekdays (sed: 71%; mod: 63% vig: 81%).

Interestingly, the reliability of SOPARC observations did not significantly change with the rounds of observation. In the case of informally organized activities, observers had better reliability when counting Asian park users engaging in informally organized activities than non-organized areas (sed:79% vs 73%; mod:73% vs 66%; vig:83% vs 78%). When counting Latinos and African Americans however, reliability was consistently lower when assessing organized activities, compared with areas with non-organized activities.

In terms of settings of the target area, reliability when observing basketball courts was similar across race ethnicities being higher when assessing sedentary (Asian:75%; Latino:74%; African Americans:76%) and vigorous activity (A:75%; L:80%; AA:77%) than moderate activity (A:65%; L:55%; AA:61%). In the case of baseball and handball court areas, reliability was significantly lower than basketball courts when counting Latinos (all physical activity intensities) and Asian Americans engaging in sedentary and moderate activity. Reliability was significantly higher in baseball-handball when assessing African Americans engaging in sedentary (91%) and moderate activity (87%). Playground areas had similar reliability as basketball courts, with the exception of significantly lower values when counting Latino and African American sedentary activity (L: 58%; AA: 65%). Reliability when observing swing areas was consistently high for Asians (sed:82%; mod:73%; vig:87%) and consistently low for Latinos (sed:64%; mod:47%; vig:71%). Finally, observing water features seemed to be harder when looking at sedentary activity than for moderate and vigorous across all groups.

## Discussion

Despite most SOPARC studies reporting high reliability and a protocol that has been widely accepted and adopted in outdoor recreation research [6, 7, 27, 29, 30], it is still important to understand how reliability of the observations might be compromised by factors such as park users characteristics being observed or contextual conditions. With SOPARC being increasingly used to assess park use behaviors and preferences in diverse communities and environments [8, 31, 32], it is even more important to understand how its reliability can change in different observation contexts. Identifying which factors cause observations to have lower reliability can help improve future SOPARC training protocols.

In this context, this study uses 4725 paired SOPARC observations in 20 New York City parks conducted during Spring and Summer 2017 to analyze the reliability and interobserver agreement of observers using SOPARC to assess race/ethnicity and physical activity in different park settings. Results concur with a large body of evidence regarding SOPARC reliability at observing park users and physical activity [4, 7, 10, 33, 34]. High levels of reliability were achieved when counting the number of people in the parks (ICC = 0.92), indicating excellent agreement beyond chance. Reliability scores however were affected by the population being observed, the physical activity level, and contextual conditions and settings of the target area at the time of the observation.

### Reliability and race/ethnicity

Despite the observation of all three targeted race/ethnicity groups –Asian American, Latino, African American- drawing high levels of reliability (ICC > 0.75), agreement between observers was harder to achieve when counting Latino park users. This was also recently observed by Banda et al. [8]. Low interobserver agreement when observing the number of Latinos in a park area can be explained by race/ethnicity being a socially constructed classification that depends both on phenotype and on their associated meanings. In our case, parks located in neighborhoods with predominantly Latino populations, saw a higher race-ethnicity mix, than parks located in Asian American Neighborhoods. The fact that Latinos and African Americans usually were found together in the same parks, while the population found in Asian American predominant parks were more homogeneously Asian, might help explain why observers had more troubles at agreeing when observing Latino and African American park users and agreed more often when classifying Asian park users.

Low reliability when trying to assess the number of Latinos in an area can also be explained by a potential two-way missclassification. From our experience during training, trying to assess the race/ethnicity of a potential Latino person from a distance often resulted in discussions between how to classify that person. Skin color plays an important role in racial and ethnic identification [35, 36], and with Latino population typically exhibiting larger intragroup phenotype variation [37], doubts on how to potentially classify Latino park users were recurrent. In informal discussions, raters mentioned particular difficulty distinguishing between Latino and White; Latino and South Asian (India, Pakistan); and Latino and African-American. Disagreements regarding the race/ethnicity of a potential Asian person were often discussed as a decision between Asian or Whiteand only in some cases between Asian-Latino. In the same way, disagreements regarding the race/ethnicity of a potential African American involved only a decision between African American and Latino and in some cases African American and White. Also noteworthy, is the fact that when in doubt, observers were instructed to default to the “other” race/ethnicity category, adding the possible scenario of the first observer having doubts on how to classify a potential Latino person and defaulting to other, and the second observer having no doubts and thus classifying them as Latino.

In any case, our findings suggest that most studies using SOPARC to identify Latino park use might actually only be analyzing the behavior of those Latinos with darker skin tones, who are more easily classified as Latinos. Given the relevant socioeconomic inequalities between light and dark Latino individuals within the same ethnic groups [37], it is important that future SOPARC studies acknowledge and address this limitation. And with the growth of Latino populations in the US and the need of designing tailored public policies towards encouraging physical activity, these findings could be valuable when designing future park-use studies. SOPARC training should incorporate specific attention on how to properly assess race/ethnicity based on phenotype characteristics. Nonetheless, these characteristics should only be applied for direct observations if race/ethnicity is an important individual variable for the study. Employing local community members in SOPARC observations has been reported as a way to help overcome some of these issues [38] although it should be noted that past studies did not find evidence of an association between observers’ demographic characteristics and better identification race/ethnicity traits [36, 39].

### Reliability and physical activity

Agreement for physical activity levels was even harder to reach than trying to identify race/ethnicity, consistent with the original reliability assessment by McKenzie et al. [33]. Our results suggest that observing sedentary and vigorous activities might be easier than moderate physical activity. It is a counterintuitive finding, as more dynamism should be harder to assess, but one that was also recently found by Santos et al. [10]. Once again this can be partially explained by a regression to the mean and the fact that moderate physical activity is adjacent to two other categories allowing for two types of misclassification (sedentary-moderate; and vigorous-moderate), while sedentary and vigorous activity are only adjacent to the moderate category. Other explaining factors are the fact that, counting immobile people (sedentary) might be easier than counting moving people or observers might tend to unconsciously fixate and focus on the more dynamic movement (vigorous) on the target area. While training provided a clear list of activities and at which level of physical activity they should be considered, it is also possible that observers would unconsciously default some activities that should be classified as vigorous into moderate. All of these factors might be contributing to explain why reliability is lower when counting moderate physical activity, and higher when assessing sedentary or vigorous physical activity, and should also be accounted for in future training.

### Reliability, contextual conditions and target area settings

Regarding contextual conditions and settings of the target area at the time of the observation potentially affecting reliability scores, an important finding of this study has been that interobserver agreement did not decay with each additional round of observation. The SOPARC protocol with modified format seems to be adequate as the quality of observations was not impacted by the amount of time spent observing or observers fatigue. Other than that, the most important contextual condition affecting interobserver reliability were the number of people present in the target area, and the type of target area that was being observed. Observers achieved a very high agreement when observing areas with five or fewer people. The type of target area for its part, also significantly affected reliability. While swing areas recorded very high interobserver reliability, basketball courts and playgrounds reached low agreement rates close to 60%.

These low reliability statistics in specific areas of the park, can be partially explained by a combination of some race/ethnicities being more difficult to assess than others, some physical activity being harder to identify, and the fact that areas with more people might be harder to assess. African American males for instance are more frequently observed engaging in vigorous physical activity in basketball courts [15]. The combination of more people in the target area engaging in a highly dynamic activity can lower interobserver reliability. Similarly, complex cognitive tasks such as identifying race/ethnicity and physical activity at the same time, can work well in calm and well-defined areas such as swings, but can prove tricky in intricate areas such as playgrounds.

### Reliability and the combination of physical activity, race and context and target area settings

Trying to assess a hard-to-measure target, such as physical activity categories that are hard to distinguish, and in the context of a difficult-to-observe target area, can substantially drop reliability values below acceptable thresholds. This variability was also described by Chung-Do et al. [40] who had agreement rates as high as 0.94 for sedentary girls, and as low as 0.44 for vigorous boys.

Our results suggest that observers might benefit from subdividing the target areas whenever more than 5 people are present, or in the case that more moderate activities are taking place. Modifying the SOPARC form to anchor observations based on race/ethnicity and age instead of sex and physical activity could also improve reliability since race and physical activity are the more difficult-to-observe variables. Whenever possible, researchers should also consider balancing the number of scans with the number of inputs to be recorded per scan. Transitioning towards more scans per observation--one scan for each sex, age, and race/ethnicity, with each scan having only to record the physical activity level—could alleviate problems associated with large counts. While some technological assistance such as iSOPARC can be valuable to streamline coding and data management [10], if future SOPARC studies want to keep increasing the amount of information gathered per scan, they should consider a technological change or accept the drawbacks of lower reliability scores. In the future, if the appropriate permits are obtained, researchers might want to start using pictures or video cameras that can provide static assessments of the conditions of the park area and its park users, which can later be examined more thoroughly by human researchers or by machine learning [41].

## Conclusions

Observational protocols such as SOPARC are key tools to understand park use to accurately assess physical activity. This study set to understand how reliability of SOPARC observation can be affected by trying to link individual characteristics such as race ethnicity and physical activity. At the same time, we were interested on testing how contextual conditions at the time of the observation and the settings of the target area could also affect SOPARC reliability. Results suggest that SOPARC is an excellent tool for assessing population level physical activity, and justified the use of the modified form, which allows for potential alternative analyses at the individual level. However, its limitations arise when trying to link observations by race, age, sex and physical activity at the same time. In particular, agreement between observers was harder to achieve when counting Latino park users, while agreement for sedentary and vigorous activities was easier than moderate physical activity. Observations in some park areas such as basketball courts and playgrounds reached agreement rates that can only be considered as low. Similarly, active areas and areas that can gather more people may benefit from simultaneous observation by multiple raters to allow more stable estimates.

Based on these results, subsequent iterations of the SOPARC protocol might want to emphasize training on the items that have shown lower reliability such as race/ethnicity. Reports of SOPARC reliability should also focus on reliability in nonempty areas, and not the complete set of observations. Alternatively, studies interested on observing specific attributes regarding physical activity, age and race/ethnicity might consider changing the form layout in order to streamline the process and improve reliability.

## Supplementary information

**Additional file 1.** Modified SOPARC coding sheet that was used by this study, which allows to link sex and age observations with race/ethnicity and physical activity.

## Data Availability

The datasets used and analyzed during the current study are available from the corresponding author on reasonable request.

## Abbreviations

A: Asian
AA: African American
ICC: Intraclass Correlation Coefficient
L: Latino
Mod: Moderate
Sed: Sedentary
SOPARC: System for Observing Play and Recreation in Communities
Vig: Vigorous
