# Supplementary material for: Use of SOPARC to assess physical activity in parks: do race/ethnicity, contextual conditions, and settings of the target area, affect reliability?
Source: BMC Public Health. 2019 Dec 23;19:1730. doi: 10.1186/s12889-019-8107-0 (PMC6929368; doi:10.1186/s12889-019-8107-0)
Supplement: Supplementary file 1 — Additional file 1. Modified SOPARC coding sheet that was used by this study, which allows to link sex and age observations with race/ethnicity and physical activity. [file 12889_2019_8107_MOESM1_ESM.pdf]

Park ID: ________________ **Date:** ____/____/________ **Day of week:** ____________ **Round:** ____________

Target area ID: ________________ **Period:** 10am 3pm 4:30pm 6pm **Observer ID:** ____________ **Start time:** ____________

Conditions of target area: Accessible Supervised Equipped Usable Organized - F / I Dark Empty

Shade cover: Predominantely shade Shade/sun mix Predominately sun **Adult role:** Not present Staff Parent/caregiver

Participants		White			Black			Asian			Latino			Other/Unsure		
**Female**	**Primary Activity**	**Sed**	**Mod**	**Vig**	**Sed**	**Mod**	**Vig**	**Sed**	**Mod**	**Vig**	**Sed**	**Mod**	**Vig**	**Sed**	**Mod**	**Vig**
**0-4 years**																
**5-10 years**																
**Teen**																
**Adult**																
**Senior**																
**Participants**		**White**			**Black**			**Asian**			**Latino**			**Other/Unsure**		
**Male**	**Primary Activity**	**Sed**	**Mod**	**Vig**	**Sed**	**Mod**	**Vig**	**Sed**	**Mod**	**Vig**	**Sed**	**Mod**	**Vig**	**Sed**	**Mod**	**Vig**
**0-4 years**																
**5-10 years**																
**Teen**																
**Adult**																
**Senior**																

Comments:

v.4 (04/20/2017)

Conditions of target area

Accessible: e.g., not locked or rented to others
Supervised: e.g., park staff or coach present
Equipped: e.g., balls available
Usable: e.g., not excessively wet, broken; for its intended purpose
Organized Formal: e.g, team sporting event
Organized Informal: e.g, groups playing a sport/game
Dark: e.g., sufficient lighting
Empty: e.g., scan area is empty

Age group

0-4 years old = dependent child, from infancy to 4 years of age, likely assisted by a caregiver
5-10 years old = independent child, from 5 to 10 years of age, unlikely to need assistance
Teen = people from 11 to 19 years of age
Adult = people from 20 to 64 years of age
Senior = people from 65 years of age or older

**Sedentary**	**Moderate/Walking**	**Vigorous**
Sitting	Standing, holding a child	Running
Standing	Pushing a child on a swing	Jumping
Lying down	Child on a swing, assisted	Cartwheels
Child being carried	Child on seesaw, assisted	Child on a swing, no assistance
	Pushing a child on a seesaw	Child on seesaw, no assistance

Activity codes

**Sedentary**	**Sports**		**Fitness**	**Active game**
Artwork	Baseball	Horseshoes	Aerobics	Climbing/sliding
Chess/checkers	Basketball	Soccer	Fitness stations	Jumping (rope, hoops, hop scotch)
Lying	Cheerleading	Tennis/racquet	Jogging/running	Manipulatives
Picnick	Dance	Tetherball	Strength	Monkey bars/hanging
Reading	Football	Volleyball	Walking	Swinging
Standing/Sitting	Gymnastics			Tag/chasing games
Using mobile devices	Handball			Using playground set
				Water feature/splash pad
